# Influence of Stress, Gender, and Minority Status on Cardiovascular Disease Risk in the Hispanic/Latino Community: Protocol for a Longitudinal Observational Cohort Study

**DOI:** 10.2196/28997

**Published:** 2021-05-06

**Authors:** Tonia Poteat, Linda C Gallo, Audrey Harkness, Carmen R Isasi, Phoenix Matthews, Neil Schneiderman, Bharat Thyagarajan, Martha L Daviglus, Daniela Sotres-Alvarez, Krista M Perreira

**Affiliations:** 1 Department of Social Medicine University of North Carolina School of Medicine Chapel Hill, NC United States; 2 Department of Psychology San Diego State University San Diego, CA United States; 3 Department of Public Health Sciences University of Miami Miami, FL United States; 4 Department of Epidemiology and Population Health Albert Einstein College of Medicine New York, NY United States; 5 Department of Population Health Nursing Science University of Illinois Chicago Chicago, IL United States; 6 Department of Psychology University of Miami Miami, FL United States; 7 Department of Laboratory Medicine and Pathology University of Minnesota Minneapolis, MN United States; 8 Institute for Minority Health Research University of Illinois College of Medicine Chicago, IL United States; 9 Department of Biostatistics University of North Carolina at Chapel Hill Chapel Hill, NC United States

**Keywords:** minority stress, cardiovascular disease, sexual and gender minorities, transgender, intersex, lesbian, bisexual, gay, Hispanic, Latino

## Abstract

**Background:**

Hispanic/Latino sexual and gender minorities (SGM) are the fastest growing ethnic group of SGM in the United States. Cardiovascular disease (CVD) is a leading cause of morbidity and mortality among Hispanics/Latinos. SGM inequities in CVD risk have been identified as early as young adulthood, and minority stress has been identified as a potential mediator. Yet, the small number of ethnic or racial minority participants in SGM studies have precluded the examination of the intersections of sexual orientation, gender identity, and race and ethnicity.

**Objective:**

Minority stress models conceptualize relationships between stressors in minority groups and health outcomes. In this study, we will (1) examine the influence of sexual orientation and gender identity on CVD risk among all *Hispanic Community Health Study/Study of Latinos* (HCHS/SOL) participants at visit 3 (2021-2024; N~9300); (2) model pathways from sexual orientation and gender identity to CVD risk through stigma, discrimination, and stress in a 1:2 matched subcohort of SGM and non-SGM participants at visit 3 (n~1680); and (3) examine the influence of resilience factors on sexual orientation or gender identity and CVD risk relationships among subcohort participants at visit 3 (n~1680).

**Methods:**

This study will leverage existing data from the parent HCHS/SOL study (collected since 2008) while collecting new data on sexual orientation, gender identity, stigma, discrimination, stress, coping, social support, and CVD risk. Data analysis will follow the SGM minority stress model, which states that excess stigma against SGM populations leads to minority stress that increases CVD risk. In this model, coping and social support serve as resilience factors that can mitigate the impact of minority stress on CVD risk. Cross-sectional and longitudinal regression models as well as structural equation models will be used to test these relationships.

**Results:**

This study was funded by the National Heart, Lung, and Blood Institute in March 2020. Recruitment is scheduled to begin in the first quarter of 2021 and continue through 2024.

**Conclusions:**

Understanding the influence of stigma-induced stress on CVD risk among Hispanic/Latino SGM has significant implications for the development of culturally specific CVD risk reduction strategies. Study findings will be used to build on identified Hispanic/Latino cultural strengths to inform adaptation and testing of family and community acceptance interventions.

**International Registered Report Identifier (IRRID):**

PRR1-10.2196/28997

## Introduction

### Background

Cardiovascular disease (CVD) is the second leading cause of death among Hispanic/Latino adults [1]. This population bears a heavy burden of obesity, diabetes, poorly controlled hypertension, and other cardiovascular risk factors [2]. Recent studies have identified heterogeneity in cardiovascular risk among Hispanic/Latino adults by heritage group, gender, acculturation, and duration of US residency [3,4]. However, variability in cardiovascular risk factors has not been systematically examined by sexual orientation or gender identity.

The inclusion of sexual orientation measures in national, population-based surveys such the National Health Interview Survey, the National Health and Nutrition Examination Survey, and the Behavioral Risk Factor Surveillance Survey (BRFSS) have provided data on the elevated prevalence of cardiovascular risk factors among sexual minorities, including smoking [5-7], obesity [6-8], high blood pressure [6], and glycosylated hemoglobin [6]. An analysis of data from the National Longitudinal Study of Adolescent to Adult Health identified sexual orientation inequities in cardiovascular risk behaviors (eg, smoking), as well as clinical measures (eg, blood pressure) and biomarkers (eg, C-reactive protein), beginning in young adulthood [9].

The BRFSS is the only national population-based survey to have published data that includes a module to assess gender identity. BRFSS data indicate transgender adults experience disparities in weight [7], smoking [10], and myocardial infarction [11]. The aforementioned studies have also identified variations in cardiovascular risk among sexual and gender minorities (SGM) by sex assigned at birth (eg, male, female), gender identity (eg, transgender woman, cisgender woman), and sexual identity (eg, gay, bisexual) [5,6,8]. However, the small number of ethnic minority SGM in these samples have precluded specific analyses within Hispanic/Latino SGM, even though Hispanic/Latino SGM are the fastest growing ethnic group of SGM in the United States [12].

### Rationale

Psychosocial stressors play an important role in CVD risk [13]. A global investigation of the determinants of CVD outcomes, in 52 countries over 7 continents, found that psychosocial stress was a powerful predictor of myocardial infarction, comparable in impact to smoking [14,15]. Baseline data from the *Hispanic Community Health Study/Study of Latinos* (HCHS/SOL) found that a composite score for psychological distress, including depressive symptomatology and trait anxiety, was significantly associated with obesity for women, diabetes for men, and smoking for all participants [16].

Stigma and discrimination are common psychosocial stressors for minority groups and are associated with a range of negative health outcomes, including increased risk for CVD [17-21]. A recent nationally representative sample of 489 SGM reported prevalent harassment or threats (57%) and violence (51%) because of their sexual orientation or gender identity [22]. The HCHS/SOL Sociocultural Ancillary Study found that most Hispanics/Latinos (80%) reported exposure to ethnic discrimination during their lifetime [23]. While exposure to ethnic discrimination varied based on education, income, and acculturation, lack of sexual orientation and gender identity measures in HCHS/SOL precluded examination by SGM status. Other research suggests that Hispanic/Latino SGM experience the unique stressors of both racism and ethnocentrism within SGM communities and rejection of their sexual orientation and/or gender identity by their Hispanic/Latino families and communities [24].

Resilient coping and social support may mitigate the impact of psychosocial stressors on CVD risk. Hispanic/Latino cultural values are hypothesized to engender strong social supports that buffer health risks [25-27]. For example, lower acculturation and foreign-born nativity, often used as proxies for stronger cultural values, are associated with lower CVD prevalence [28]. However, SGM Hispanic/Latino individuals may lose access to these cultural buffers if they are rejected by their families or communities, or forced to conceal their sexual orientation or gender identity [24]. Connection to SGM community may mitigate minority stress for Hispanic/Latino SGM [29,30]. However, whether this connection reduces CVD risk for Hispanic/Latino SGM has not been studied.

### Conceptual Framework

This study, *Stress Gender and Minority Status in the Study of Latinos* (SGM SOL), aims to advance scientific knowledge of how stigma at the intersection of ethnic identity, sexual orientation, and gender identity impacts CVD outcomes for Hispanic/Latino SGM. It draws on two complementary conceptual frameworks: intersectionality theory and the minority stress model. Intersectionality addresses how the stress of holding multiple stigmatized identities, such as being a racial/ethnic minority, SGM, and/or an immigrant, may compound inequities [31,32]. Intersectionality theory guided our decision to measure multiple social identities including sexual orientation and gender identity, socially ascribed race, and immigration background. In keeping with a fundamental tenet of intersectionality theory—that social identities are mutually constituted—we plan to use adapted measures of stigma, discrimination, and social stress that are inclusive of multiple simultaneous identities without requiring participants to attribute experiences to a particular identity [33]. The minority stress model posits that stigma and discrimination create excess minority stress for SGM populations that result in health inequities, and that these inequities can be mitigated by resilience factors, such as positive coping and social support [34-36].

**Figure 1:**
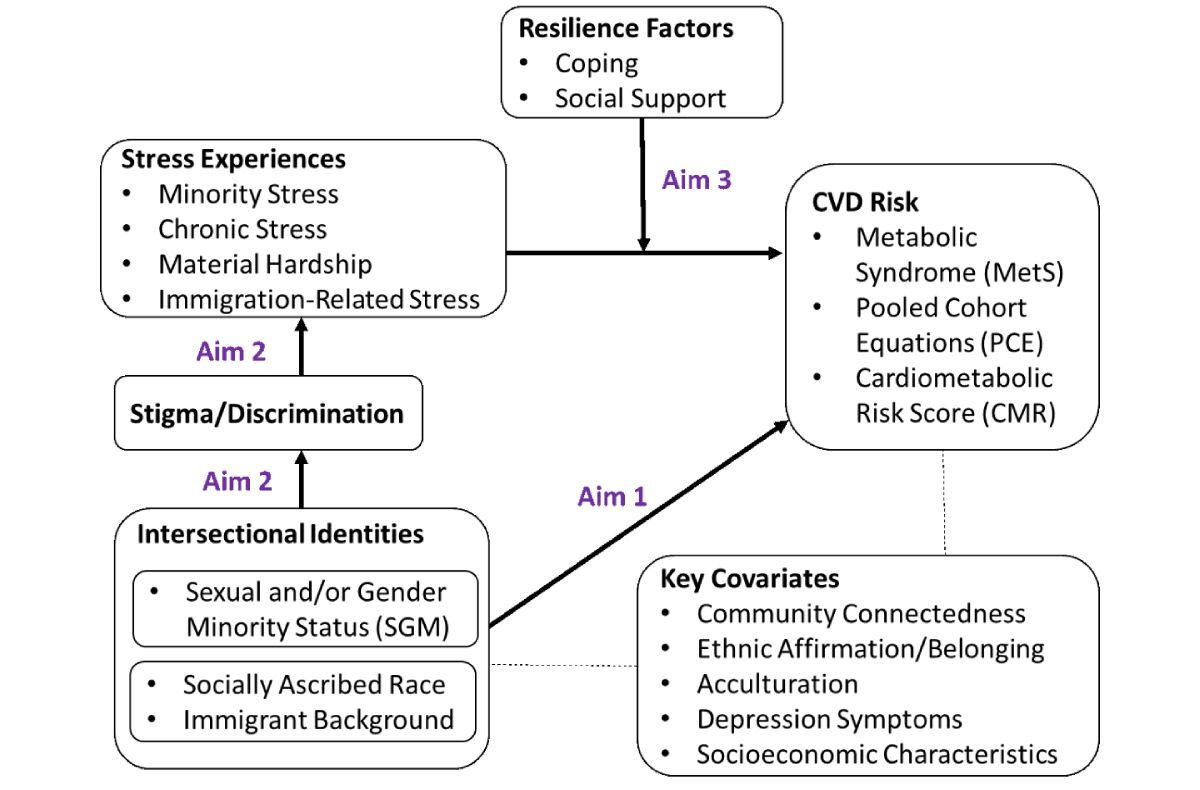
Conceptual framework and study aims for the SGM SOL (Stress Gender and Minority Status in the Study of Latinos) study. CVD: cardiovascular disease.

SGM SOL aims to assess relationships among CVD risk and stigma, discrimination, stress, and coping and social support by comparing SGM with non-SGM Hispanic/Latino adults, thereby testing relationships theorized by the minority stress model, as depicted in Figure 1.

### Objectives

The primary aims of this study are to (1) examine the influence of sexual orientation or gender identity on CVD risk during visit 3 of HCHS/SOL (N~9300); (2) model pathways from sexual orientation or gender identity to CVD risk through stigma, discrimination, and stress in an HCHS/SOL subsample (n~1680); and (3) examine the influence of resilience factors on sexual orientation or gender identity and CVD risk relationships in the HCHS/SOL subsample (n~1680).

## Methods

### Setting

SGM SOL builds on the infrastructure of HCHS/SOL, a multicenter, longitudinal, observational cohort study designed to evaluate prevalence, incidence, and risk and protective factors for cardiovascular disease and other chronic conditions among Hispanic/Latino adults in the United States. It is the largest well-characterized longitudinal cohort study of diverse self-identified US Hispanic/Latinos with 16,415 adults (aged 18-74 years) enrolled at baseline. Using a 2-stage probability sample, households and participants were randomly selected using stratification and oversampling at each stage in 4 US sites—the Bronx, NY; Chicago, IL; Miami, FL; and San Diego, CA [37]. Full details about the study design have been previously published [37,38].

### Study Design and Population

SGM SOL is a longitudinal observational cohort study of HCHS/SOL adults who will participate in visit 3. HCHS/SOL collected in-person data in 2008-2011 (visit 1), and 81% of eligible participants (n=11,623) returned for visit 2 (2014-2017). We expect 80% of the visit 2 participants to return for visit 3. Thus, we project approximately 9300 visit 3 participants. Based on national data, which indicate that 6.1% of the Hispanic/Latino population in the United States identify as SGM [39], we anticipate that 560 HCHS/SOL participants will meet at least one of the SGM inclusion criteria.

#### Inclusion Criteria

All visit 3 participants are asked 4 screening questions pertaining to sexual orientation or gender identity: (1) sex assigned at birth; (2) current gender identity, including the age at which gender identity first differed from sex assigned at birth, if applicable; (3) any history of an intersex condition; and (4) any history of same-sex attraction, including age of first same-sex attraction, if applicable. SGM status is operationalized as reporting a current gender that is different from one’s sex assigned at birth, reporting any history of an intersex condition, or reporting any history of same-sex attraction.

### Study Procedures

**Figure 2:**
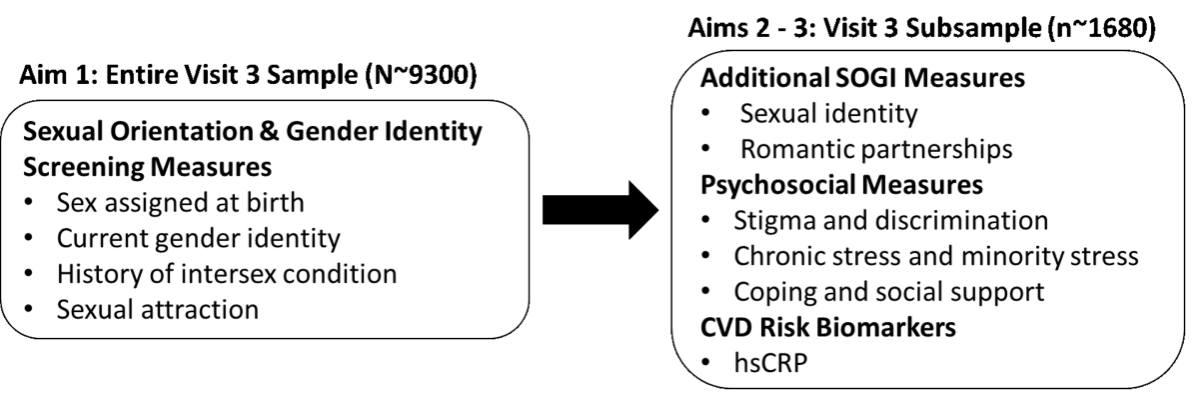
SGM SOL (Stress Gender and Minority Status in the Study of Latinos) measures added to the core HCHS/SOL (Hispanic Community Health Study/Study of Latinos) measures in visit 3, by aim. SOGI: sexual orientation or gender identity, CVD: cardiovascular disease, hsCRP: high-sensitivity C-reactive protein.

As shown in Figure 2, the first aim will be addressed using the entire HCHS/SOL visit 3 cohort (N~9300) who complete the screening measures related to sexual orientation or gender identity. The second and third aims will be addressed by enrolling 560 SGM and 1120 non-SGM who are matched 2:1 to the SGM participants by site, age, and sex assigned at birth (n~1680). All study procedures have been approved by the central institutional review board (IRB) at the University of North Carolina Chapel Hill and by the local IRBs of all participating institutions.

#### Recruitment

Recruitment for the SGM SOL cohort is being coordinated with the existing recruitment activities for HCHS/SOL visit 3. At the end of their scheduled visit 3 assessment, all current participants who screen as SGM are invited to participate in SGM SOL. A matched subsample of participants who screen as non-SGM are invited to participate at the end of their visit 3 assessment. Matching is based on sex assigned at birth (female, male), age (<50, 50-59, ≥60 years), and site (the Bronx, Chicago, Miami, San Diego). An algorithm embedded within the HCHS/SOL visit 3 electronic data management system indicates whether a participant should be invited to take part in the SGM SOL ancillary study. All individuals who agree to participate in SGM SOL will complete the informed consent process.

#### Data Collection

At visit 3, all participants complete the 4 SGM screening questions and psychosocial questionnaires and have a fasting blood sample and anthropometry measures taken. All HCHS/SOL questions are asked in the participant’s language of preference. Questionnaires are administered by bilingual staff who are centrally trained and certified in interviewing techniques. The SGM SOL matched cohort subsample completes an additional 30-minute structured interview and laboratory testing for high-sensitivity C-reactive protein. The structured interview includes additional questions on sexual orientation and gender identity as well as detailed questions regarding stigma, discrimination, stress, and coping and social support. Individuals who are interested in participating but unable to do so at the time of their visit 3 assessment are given the option to complete the SGM SOL interview via phone at a later date.

### Measures

All new SGM SOL study measures are listed in Table 1, organized by visit and topic area. We also list related measures for visit 3 or previously collected at visits 1 or 2. All HCHS/SOL visit 3 participants will complete the measures listed in Table 1 in the visit 3 column as well as the 4 SGM screening questions. The SGM SOL study participants will complete the additional measures listed in Table 1 in the new measures column. Except where noted below, all study instruments have existing, validated Spanish translations. In the few cases where translations were not available, items were translated into Spanish by culturally knowledgeable bilingual staff, then back-translated by different culturally knowledgeable bilingual staff members to ensure accuracy [40]. Below, we describe each of the main measures listed in the conceptual framework.

**Table 1:** SGM SOL (Stress Gender and Minority Status in the Study of Latinos) measures and related HCHS/SOL (Hispanic Community Health Study/Study of Latinos) measures at visits 1, 2 and 3.

Constructs	New SGM SOL measures	HCHS/SOL measures		
		Visit 3 (2020-2023)	Visit 2 (2014-2017)	Visit 1 (2008-2011)
Sexual and/or gender minority screening measures	Sex assigned at birth	—^a^	Interviewer-ascribed sex	Interviewer-ascribed sex
	Current gender identity, age when gender identity differed from sex assigned at birth, if applicable	—	—	—
	Intersex/DSD^b^	—	—	—
	Sexual attraction, age of first same-sex attraction, if applicable	—	—	—
Additional sexual orientation measures	Sexual identity, including age of first sexual minority identity, if applicable	—	—	—
	Romantic partners, age of first same-sex partnership, if applicable	—	—	—
Race and immigration background	“Street” race (ie, race ascribed by others)	—	US citizenship, immigrant visa status	Self-reported race, Hispanic/Latino background, place of birth, parents’ place of birth
	—	Years in the United States	Years in the United States	Years in the United States
Stigma and discrimination	Identity stigma (adapted for all identities)	—	—	—
	Everyday Discrimination Scale - short form (adapted for all identities)	—	—	—
Stress experiences	Minority Stress - Rejection Anticipation subscale (adapted for all identities)	—	—	—
	Chronic stress	—	Chronic stress	—
	Material hardship	—	—	—
	Immigration-related stress	—	—	—
Resilience factors	Brief Resilient Coping Scale	—	—	—
	Interpersonal support evaluation list	—	Interpersonal support evaluation list	—
CVD^c^ risk	PCE^d^: sex assigned at birth, age, street race, total cholesterol, HDL-C^e^, SBP^f^, hypertension treatment, type 2 diabetes, and tobacco use	Tobacco use; medications for hypertension, hyperlipidemia, and/or diabetes	Tobacco use; medications for hypertension, hyperlipidemia, and/or diabetes	Tobacco use; medications for hypertension, hyperlipidemia, and/or diabetes
	MetS^g^: waist circumference, blood pressure, triglycerides, HDL-C, fasting glucose, medications for hypertension, hyperlipidemia, and/or diabetes	Waist-hip ratio, blood pressure	Waist-hip ratio, blood pressure	hsCRP^h^, waist-hip ratio, blood pressure
	CMR^i^: blood pressure, glycosylated hemoglobin, hsCRP, waist circumference	Total cholesterol, HDL-C, LDL-C^j^, triglycerides, fasting glucose	Total cholesterol, HDL-C, LDL-C, triglycerides, fasting glucose	Total cholesterol, HDL-C, LDL-C, triglycerides, fasting glucose
Potential confounders and covariates	LGBT^k^ Minority Stress Scale - Community Connectedness subscale (adapted for all)	Years in the United States	Years in the United States	Years in the United States
	Multigroup Ethnic Identity Measure - Affirmation and Belonging subscale	—	Short Acculturation Scale for Hispanics	Short Acculturation Scale for Hispanics
	—	Income, employment, education, marital status	Income, employment, education	Income, employment, education, occupation, marital status
	Hormone use, including for gender affirmation	—	Female hormone use	Female hormone use

^a^Not applicable.

^b^DSD: differences of sex development.

^c^CVD: cardiovascular disease.

^d^PCE: pooled cohort equation.

^e^HDL-C: high-density lipoprotein cholesterol.

^f^SBP: systolic blood pressure.

^g^MetS: metabolic syndrome.

^h^hsCRP: high-sensitivity C-reactive protein.

^i^CMR: cardiometabolic risk score.

^j^LDL-C: low-density lipoprotein cholesterol.

^k^LGBT: lesbian, gay, bisexual, and transgender.

#### Exposure Variables

The National Institutes of Health define SGM populations as “individuals who identify as lesbian, gay, bisexual, asexual, transgender, Two-Spirit, queer, and/or intersex. Individuals with same-sex or -gender attractions or behaviors and those with a difference in sex development are also included” [41]. Several best practice recommendations for measuring sexual orientation and transgender-inclusive gender identity have been published [42]. The Gender Identity in US Surveillance (GenIUSS) group recommends the 2-step method of measuring sex assigned at birth and current gender identity separately [43]. No consensus exists on measurement of intersex status and no federal surveys include it [44]. The Sexual Minority Assessment Research Team recommends assessment of 3 dimensions of sexual orientation: attraction, behavior, and identity [45] (Table 1).

##### SGM Screening Measures Among All Visit 3 Participants

In addition to the GenIUSS group, the Center of Excellence for Transgender Health [43] recommends a 2-step measure to ascertain transgender status. The measure has been validated with a variety of populations in both English and Spanish [46-49]. The items include (1) “What sex were you assigned at birth, meaning on your original birth certificate? (a) Male, (b) Female;” and (2) “What is your current gender identity? (a) Male, (b) Female, (c) Transgender Male, (d) Transgender Female, (e) Gender nonbinary, (f) Some other identity.” To measure intersex status, we ask, “Have you ever been told by a doctor that you have a difference of sexual development or an intersex condition?” This item is adapted from a measure recommended by the Williams Institute and tested online [43,44]. To measure sexual attraction, we ask, “People are different in their sexual attraction to other people. Which best describes your feelings? Are you attracted (a) Only to females, (b) Mostly to females, (c) About equally often to males and females, (d) Mostly to males, (e) Only to males, (f) I have never felt sexually attracted to anyone at all.” This item has been used in multiple national surveys in English and Spanish [50].

##### Additional Sexual Orientation Measures Among the Ancillary Study Subsample at Visit 3

Sexual orientation identity is measured using an item that has been validated in a variety of populations in English and Spanish across multiple federal population-based surveys [49-51]. The item reads, “Do you think of yourself as (a) Not gay or lesbian, that is straight; (b) Gay; (c) Lesbian; (d) Bisexual; (e) Something else.” Sexual orientation behavior is measured using a modified item from the General Social Survey [50] that reads, “In your lifetime, have your romantic partners been (a) Males only, (b) Females only, (c) Males and Females, (d) I have not had romantic relationships.”

##### Race and Immigration Background

We measure socially ascribed race, that is, the race that is usually ascribed to the participant by others, using 2 items previously used in the Latino National Health and Immigration Survey and the Pew National Survey of Latinos [52]. The first measures self-reported skin color, a physical characteristic often used to ascribe race to individuals. The second measures self-reported “street race” or what race participants think others attribute to them based on their appearance. These measures have been shown to predict inequities in health status, regardless of self-identified race [53]. Immigration background was ascertained at visit 1 where participants identified their country of birth and their parents’ country of birth. Respondents provided their self-reported Hispanic/Latino background as Cuban, Dominican, Mexican, Puerto Rican, Central American, South American, mixed, or other.

#### Potential Mediators

##### Stigma and Discrimination

We measure stigma using the Identity Stigma Scale, which asks respondents to agree or disagree along a 4-point Likert scale to a series of 6 questions beginning with the stem, “These next statements refer to ‘a person like you’; by this I mean persons who have the same gender, race, sexual orientation, nationality, ethnicity, and/or socioeconomic class as you. I would like you to respond on the basis of how you feel people, in general, regard you in terms of such groups” [54]. Response options include items such as “Most people believe a person like you cannot be trusted” and “Most people look down on people like you.” Previously, HCHS/SOL included 2 items measuring perceived discrimination of Hispanic/Latinos adopted from Gil et al’s Acculturative Stress Index [55]. The Everyday Discrimination Scale is a widely used measure that assesses experiences of discrimination, and the short form has been used in the Chicago Community Adult Health Study [56]. The benefit of both measures is that they have been adapted to apply to stigma and discrimination experiences for any identity without requiring the respondent to attribute the experience to any specific identity or preset list of identities, consistent with intersectionality theory. Lastly, we include one additional question adapted from the BRFSS Reactions to Race Module to measure discrimination in a health care setting [57].

##### Stress Experiences

Minority stress is measured using a 6-item adaptation of the Rejection Anticipation subscale of the LGBT Minority Stress Scale [58]. To make this scale applicable to all participants while focusing on stress related to sexual orientation or gender identity, we replaced “because I am LGBT” with “because of my sexual orientation or gender identity” in the stem of the question. The scale includes questions such as, “I stay on guard and alert because something bad might happen to me because of my sexual orientation or gender identity.” Participants respond using a 5-point Likert scale. The 8-item Chronic Burden Scale was assessed at visit 2 [59] and is being reassessed among the ancillary study subsample as a measure of chronic stress [60]. Using established scales available in Spanish and English, material hardship [61] and immigration-related stress [62] are measured as forms of stress unrelated to sexual orientation or gender identity.

#### Moderators

##### Resilience Factors

Coping is measured using the 4-item Brief Resilient Coping Scale [13] in which participants use a 5-point Likert scale to report how well each item describes their actions. Social support is measured by the Interpersonal Support Evaluation (ISEL) [63,64]. The ISEL contains 12 items, available in English and Spanish, which assess the perceived availability of social support on a 4-point scale ranging from “definitely false” to “definitely true.” All items are summed to yield a total score (range 0-36). Longitudinal assessment is possible since ISEL was also measured during visit 2.

#### Key Covariates

Community connectedness may affect both stigma and minority stress [30,65,66]. For example, individuals who are connected to marginalized communities may experience and/or have more awareness of stigma. The same individuals may also experience less stress because their connections allow for access to greater social support. We measure community connectedness using a subscale of the LGBT Community Connectedness scale adapted for all identities. For example, we replaced “I feel connected to other LGBT people” with “I feel connected to other people who share my sexual orientation or gender identity.” Participants agree or disagree with items along a 5-point Likert scale. To be consistent with intersectionality theory, we also measure connection to ethnic minority communities using the Affirmation and Belonging subscale of the Multigroup Ethnic Identity Measure [67]. In this measure, participants agree or disagree along a 4-point Likert scale with items such as, “I have a strong sense of belonging to my own ethnic group.” Because acculturation has known effects on CVD risk [27,68,69], and we hypothesize that it may also be related to SGM stigma, we are assessing the number of years participants have lived in the United States. The HCHS/SOL already collects measures of additional socioeconomic characteristics such as education, employment, income, occupation, and marital status.

#### CVD Risk Outcome Measures

CVD risk is multifactorial, including risk behaviors (eg, smoking), risk biomarkers (eg, blood pressure), and other risk factors (eg, diabetes). SGM SOL aggregates risk behavior, biomarkers, and other factors to assess CVD risk, operationalized as the following three outcomes: (1) the American College of Cardiology Pooled Cohort 10-Year Atherosclerotic Cardiovascular Disease Risk Assessment Equation (PCE) [70], (2) the modified International Diabetes Federation Metabolic Syndrome (MetS) [71], and (3) the cardiometabolic risk score (CMR) [72]. We outline each measure and the rationale for inclusion below.

The PCE uses a complex algorithm to estimate the risk of heart attack or stroke over the following 10 years for adults aged 40-79 years [70,73,74]. The measures used to calculate this risk include sex assigned at birth, age, socially ascribed race, total cholesterol, high-density lipoprotein cholesterol (HDL-C), systolic blood pressure (SBP), hypertension treatment, type 2 diabetes, and tobacco use. We include the PCE measure for all participants because it is commonly used in clinical practice and is likely to have real-world applicability. Consistent with prior clinical [75] and research use [76], the PCE will be operationalized as a dichotomous variable in which participants with PCE score >7.5% will be considered to have high CVD risk.

The MetS is a cluster of anthropometric, hemodynamic, and metabolic disturbances that have been associated with CVD morbidity and mortality [77]. Our assessment is similar to the International Diabetes Federation [71] and consistent with prior HCHS/SOL studies that defined MetS as meeting 3 or more of the following criteria: (a) waist circumference ≥102 cm in men and ≥88 cm in women; (b) blood pressure ≥130 mm Hg SBP and/or ≥85 mm diastolic blood pressure (DBP) or treatment for previously diagnosed hypertension; (c) triglycerides ≥150 mg/dL or treatment for this lipid abnormality; (d) HDL-C <40 mg/dL in men and <50 mg/dL in women or treatment for this lipid abnormality; and (e) fasting glucose ≥100 mg/dL or previous type 2 diabetes diagnosis [2]. We use this measure in order to compare outcomes with prior HCHS/SOL studies that use this measure [2,78,79].

The CMR is designed to characterize overall functioning across multiple measures of cardiovascular risk [72], similar to the MetS and consistent with concepts of allostatic load [72]. To compare to prior studies among sexual minorities, we are creating a cumulative biological risk score by counting the number (range 0-5) of biological markers that meet a clinically defined high-risk criterion [21]. The criteria for high risk are defined as: (a) SBP ≥140; (b) DBP ≥90 mmHg; (c) glycosylated hemoglobin ≥6.4%; (d) C-reactive protein ≥3 mg/dL; and (f) waist circumference ≥102 cm for men and ≥88 cm for women. Individuals will receive a value of 1 if they are above the risk threshold. While this measure is similar to the MetS, we include it because it has been used to examine sexual orientation disparities among young adults and may be more appropriate for the <50 years age group than traditional CVD measures [9,21]. We are analyzing the CMR only as a continuous variable since there is no established cut-off value for high risk [80].

### Data Management, Quality Assurance, and Quality Control

Each site recruits, consents, and collects data for approximately 420 participants (140 SGM and 280 non-SGM) for the SGM SOL ancillary study. Data collected at the sites and entered into the web-based data management system are identified by participant and staff ID numbers. Measures taken to ensure the security of the data include, but are not limited to requiring valid IDs and passwords to access the web-based data management system, using a firewall and user logins to shield the local area network from web users, and using secure sockets layer standard to provide encryption and user authentication. The data management system includes procedures to support efficient, high-quality data acquisition, tracking, and quality assurance. When there is active data collection, the Coordinating Center prepares monthly reports to track enrollment and data quality.

### Staff Training

Site personnel completed central training on sexual and gender minority cultural awareness, SGM SOL research protocols, and data collection instruments. Since collection of data on sexual orientation and gender identity was new for HCHS/SOL staff, we made every effort to raise cultural awareness and ensure comfort with the questions. Site principal investigators and all site personnel were invited to an SGM cultural awareness training that included a discussion of concepts and terminology related to sexual orientation or gender identity. The interactive training included opportunities to match terms with definitions, discuss relevant case scenarios, and ask questions in a judgement-free confidential manner. More than 50 personnel completed this training. This training was recorded and made available on the study website for future refresher training or to train new staff. Subsequently, personnel engaged in data collection or supervision completed study-specific training to increase familiarity with the questions, model appropriate ways to respond to potential participant questions, and provide opportunities to practice.

### Statistical Analysis

#### General Approach

The complex sampling design and sampling weights specific to this study will be incorporated into the final analyses using SUDAAN (RTI International), Stata (StataCorp), and Mplus (Muthén & Muthén). Skewed variables will be log or square root transformed for modeling, and we will account for the matched study design for aims 2 and 3 in the analysis. Variables with a substantial number of missing values (ie, >5%) will be explored to determine if they are associated with any exposure or outcome variables. If we find more than 10% missing data, we will use multiple imputation including auxiliary information about the missingness. In addition, we will conduct a series of sensitivity analyses to evaluate the robustness of conclusions drawn from the primary models to departures from missing at random assumption by comparing the magnitude of the primary effect. All models will assess for linearity of covariates and use polynomials in case of departure. We will specify two-sided tests and .05 significance level.

#### Analyses for Aim 1

Once data collection is complete, we will use logistic regression models to determine if SGM have higher prevalence of CVD risk than non-SGM Hispanic/Latino adults at visit 3 (N~9300). We will estimate adjusted prevalence rates [81] for PCE and MetS and its ratios comparing SGM versus non-SGM. Linear regression models will be used to estimate and compare the mean CRM between SGM and non-SGM. All models will be stratified by sex assigned at birth and control for age, Hispanic/Latino background, site, and key covariates listed in Table 1, including key intersectionality covariates—immigration background and socially ascribed race. If sample sizes allow, rather than dichotomize by SGM and non-SGM, we will conduct the primary analyses (PCE, MetS, and CMR) by gender identity and by sexual orientation, separately.

In order to determine if SGM have a greater increase over time in CVD risk than non-SGM adults, we will use generalized estimating equations and available repeated measures (visits 1, 2 and 3). We will model mean values for each outcome separately (PCE risk score, MetS count score, and CRM count score) over time by including SGM and age, and test the effect of SGM while adjusting for site, Hispanic/Latino background, and key covariates from Table 1. We will stratify by sex assigned at birth and include an interaction term between age and SGM to test whether the patterns of change over time are the same for SGM and non-SGM. A logit link function in the GEE model will be used for binary outcomes (PCE and MetS). We will conduct exploratory analyses by gender identity and sexual orientation if sample size allows.

#### Analyses for Aim 2

To determine if SGM status has significant indirect effects on CVD risk via stigma, discrimination, and stress, we will fit models for each continuous outcome score (PCE, MetS, CRM) separately among the SGM SOL subsample (n~1680). First, using linear regression models we will test whether (1) stress, stigma, and discrimination differ by SGM status; (2) the effect of stress, stigma, and discrimination (measured as separate constructs) on CVD risk is significant; and (3) the total effect of SGM status on CVD risk is significant. We will then use structural equation models to estimate the direct effect of SGM status on continuous CVD risk outcome scores (ie, PCE, MetS, CRM) and the indirect effects through stress, stigma, and discrimination, separately. The structural part of the model will have the outcome regressed on SGM status, one variable at a time (stress, stigma, and discrimination) and other covariates. For stress, the measurement part of the model will include 4 scales (chronic stress, minority stress, material hardship, and immigration-related stress). If sample size allows, we will conduct analyses by sex assigned at birth (male vs female), gender identity (cisgender vs transgender/nonbinary) and sexual orientation (lesbian, gay, bisexual, heterosexual).

#### Analyses for Aim 3

We will use linear regression models to test whether the effect of minority stress on continuous CVD risk outcomes is modified by (1) high resilient coping (brief scale >17) and (2) high social support (ISEL-12 >17). Models will include separate interaction terms for minority stress × coping and minority stress × social support controlling for covariates described above (and not including SGM status as the main effect). To derive the model, we will first determine the smallest number of covariates to control in the model by assessing their effects on the association of minority stress with CVD risk outcomes. We will retain covariates in the model when the change in the regression coefficient for minority stress is larger than 10%. Then, we will fit the model including the interaction term; we will consider *P*<.10 as evidence of interaction in each model. We will repeat the primary analyses stratifying by SGM to explore whether minority stress modifies the association in SGM and non-SGM, and whether these effect modifications are different.

### Sample Size

The aim 1 analysis will include the entire visit 3 sample (N~9300) with approximately 560 meeting our criteria for SGM. With the 1:2 match, 560 SGM and 1120 non-SGM participants will be available for analyses of aims 2 and 3. To ensure adequate power, we have conducted power analyses for aims 2 and 3 using a smaller sample size of 1125 (375 SGM and 750 non-SGM). Previous HCHS/SOL ancillary studies have successfully recruited >70% of eligible participants and the lower bound for our sample of SGM participants (n=375) represents 67% of likely SGM participants in HCHS/SOL visit 3.

### Power

For aim 1, we assume a total sample size of 9300 with 375 SGM participants (most conservative), power=0.8, variance inflation factor=2, and α=.05. For stratified analysis, we focus on our smallest subgroup (males) who comprise 36% of the sample. Based on these conservative assumptions, the power in both overall and stratified analyses is over 0.8 to detect prevalence ratios as small as 1.2 given a CVD risk factor prevalence ranging from 35% for the MetS to 63% for the PCE and as low as 20% for some age-sex subgroups [2,3]. For stratified analyses, the minimum detectable mean is 0.35 SD among males. The power is over 0.8 to test for each outcome (PCE, MetS, CRM) separately whether the mean is different by SGM and whether the patterns of change over time are the same.

For aim 2, power analysis was performed in Mplus using Monte Carlo simulation (10,000 replicates) to test separately the mediated effect of stigma, discrimination, and stress scores between SGM and CVD risk factor z-scores, given a conservative sample size of 1125, α=.05, and different effect sizes of direct paths based on Cohen’s guidelines for R^2^ (amount of explained variance in the outcome) [82]. We defined small, medium, and large effect sizes as 0.02, 0.13, and 0.26, given that we do not have estimates of the size of the indirect effects from the literature. We assumed the following parameters based on the literature [23,58,82]: minority stress (mean 2.08, SD 6.6 in Hispanics), discrimination (mean 1.5, SD 0.1), correlations between the SGM and CVD risk factors z-score ranging from 0.1 to 0.4, and residual variances of 0.95. The power is at least 0.9 for each effect (total, total indirect, and direct) from SGM to the CVD risk factors z-scores.

For aim 3, we performed a Monte Carlo simulation study in SAS to determine the minimum interaction term (in SDs) between high social support (ISEL-12 >17) and minority stress (continuous) on CVD risk factors z-scores assuming a conservative sample size of 1125, power=0.8, and α=.05. We generated 1000 samples and assumed the following parameters based on the literature [13,58,63]: minority stress (mean 2.08, SD 6.6 in Hispanics) and social support (range 0-36, mean ISEL-12 total 25.9, SD 6.6). The power is greater than 0.8 for an interaction term as small as 0.3 SD. Similarly, for resilient coping (range 4-20, mean 14.81, SD 2.95), the power is greater than 0.8 for an interaction term between high resilient coping and minority stress as small as 0.2 SD.

## Results

This study was funded by the National Heart, Lung, and Blood Institute in March 2020. However, in-person examinations were paused due to the COVID-19 pandemic. Recruitment for SGM SOL is anticipated to begin in the first quarter of 2021 and continue through 2024.

## Discussion

### SGM SOL

Despite the heavy impact of CVD in Hispanic/Latino communities and the data that Hispanic/Latino populations are more likely to report being SGM, remarkably little research has focused on CVD in the Hispanic/Latino SGM population [83]. Leveraging the infrastructure of the existing HCHS/SOL cohort, SGM SOL will examine relationships among stigma, discrimination, minority stress, and CVD in this population [84]. Specifically, SGM SOL will test whether there are significant associations between minority stress and CVD and whether coping and social support mitigate the negative impact of minority stress, taking into consideration acculturation. SGM SOL will contribute to knowledge about the effect of psychosocial factors on cardiovascular health and advance scientific knowledge on how intersectional stigma and discrimination become embodied as health inequities.

SGM SOL is the first CVD study of this size in the fastest growing SGM ethnic group in the United States. While most existing studies of minority stress and CVD use cross-sectional designs [85], SGM SOL captures cross-sectional and retrospective longitudinal data, strengthening the ability to make causal inferences about the nature of stress-health relationships and the impact of coping and social support on these outcomes, providing key data for future interventions. Implementation of SGM SOL provides a template for low burden measurement of SGM status in other population-based studies as well as strategies for ensuring data collection staff are comfortable collecting data with multiply marginalized populations.

Understanding the influence of stigma-induced stress as well as resilient coping on CVD risk among Hispanic/Latino SGM adults has important implications for efforts aimed at improving health in this growing minority population. These advances in our understanding are key to our ability to identify ways clinical providers can more effectively tailor their care to meet the needs of Latino/Hispanic SGM populations. Additionally, results can be used to develop family and community interventions that reduce SGM-related stigma and build on identified Hispanic/Latino cultural strengths by adapting and testing family acceptance strategies [86] that have been only been tested among youth to date.

### Limitations

It is possible that we will have fewer SGM participants than anticipated; therefore, we powered the study based on highly conservative estimates of the sample size. Maximizing sample size and power, aim 1 utilizes the full visit 3 HCHS/SOL cohort and will allow for exploratory analyses among specific SGM groups if the size of the SGM population is sufficiently large. Although HCHS/SOL is an excellent platform for a population-based study of Hispanic/Latino SGM health, the number of questions on sexual orientation and gender identity we could include for the entire cohort were limited by the need to reduce the burden on participants who are already completing 120 minutes of core data collection activities as part of visit 3. However, we will have more detailed information on sexual orientation and gender identity for all participants in the subsample. Hypothesized mediators (ie, stigma, discrimination, and minority stress) will be measured for the first time at visit 3, limiting the ability to assess temporality. However, CVD risk measures will be available for all 3 visits and measures of chronic stress for 2 visits. By assessing age at which SGM participants identified their current sexual orientation and gender identity, we will be able to assess temporality for relationships between SGM status, chronic stress, and CVD outcomes.

## Appendix

Multimedia Appendix 1 Peer-review report by the Health Disparities and Equity Promotion Study Section (National Institutes of Health).

## Abbreviations

BRFSS: Behavioral Risk Factor Surveillance Survey
CMR: cardiometabolic risk score
CVD: cardiovascular disease
DBP: diastolic blood pressure
GenIUSS: Gender Identity in US Surveillance
HCHS/SOL: Hispanic Community Health Study/Study of Latinos
HDL-C: high-density lipoprotein cholesterol
IRB: institutional review board
ISEL: Interpersonal Support Evaluation
MetS: metabolic syndrome
PCE: pooled cohort equation
SBP: systolic blood pressure
SGM: sexual and gender minorities
SGM SOL: Stress Gender and Minority Status in the Study of Latinos
