# Supplementary material for: Influence of Stress, Gender, and Minority Status on Cardiovascular Disease Risk in the Hispanic/Latino Community: Protocol for a Longitudinal Observational Cohort Study
Source: JMIR Res Protoc. 2021 May 6;10(5):e28997. doi: 10.2196/28997 (PMC8138714; doi:10.2196/28997)
Supplement: Multimedia Appendix 1 [file resprot_v10i5e28997_app1.pdf]

SUMMARY STATEMENT
(Privileged Communication)

PROGRAM CONTACT:
JOHN Kunz
301-594-0765
john.kunz@nih.gov

Release Date: 07/14/2019
Revised Date:

Application Number: 1 R01 HL149778-01

Principal Investigators (Listed Alphabetically):

PERREIRA, KRISTA M
POTEAT, TONIA C. (Contact)

Applicant Organization: UNIV OF NORTH CAROLINA CHAPEL HILL

Review Group: HDEP
Health Disparities and Equity Promotion Study Section

Meeting Date: 06/20/2019
Council: OCT 2019
Requested Start: 09/01/2019
RFA/PA: PA19-056
PCC: HHEF N
Dual IC(s): MD

Project Title: Cardiovascular Health of Sexual and Gender Minorities in the Hispanic Community Health Study/Study of Latinos (SGM HCHS/SOL)
SRG Action: Impact Score:21 Percentile:6
Next Steps: Visit https://grants.nih.gov/grants/next_steps.htm
Human Subjects: 30-Human subjects involved - Certified, no SRG concerns
Animal Subjects: 10-No live vertebrate animals involved for competing appl.
Gender: 1A-Both genders, scientifically acceptable
Minority: 1A-Minorities and non-minorities, scientifically acceptable
Age: 3A-No children included, scientifically acceptable

[redacted]

1R01HL149778-01 POTEAT, TONIA

RESUME AND SUMMARY OF DISCUSSION: The application proposes to examine the influence of sexual and gender minority (SGM) status on cardiovascular disease (CVD) risk as an ancillary study to an existing cohort study of Hispanic adults. The timely and moderately innovative examination of pathways including stigma and stress on CVD outcomes may inform evidence-based community interventions to address disparities among at-risk adults. This is a highly significant area of inquiry with a rigorous examination of prior research regarding SGM disparities. The study builds on prior work by a strong and complementary team of investigators. The rigorous study design includes clearly described biomarkers and metabolic measures in an approach appropriately guided by a conceptual framework. Minor weaknesses include the lack of clarity regarding the conceptualization of SGM identity. Some reviewers questioned whether the category will potentially mask heterogeneity in identity and behavior. Others found the justification to examine any aspect of sexual attraction as appropriate. Alternative strategies for data collection are not addressed. Overall, the strengths outweigh minor weaknesses and reviewers were enthusiastic that the application has potential for high scientific impact on CVD disparities among sexual and gender minority adults.

DESCRIPTION (provided by applicant): Hispanic/Latino sexual and gender minorities (SGM) are the fastest growing ethnic group of SGM in the U.S. Cardiovascular disease (CVD) is a leading cause of morbidity and mortality among Hispanic/Latinos. SGM inequities in cardiovascular disease (CVD) risk have been identified as early as young adulthood; and minority stress has been identified as a potential moderator. Yet, small numbers of ethnic minority participants in SGM studies have precluded examination of the intersections of sexual orientation, gender identity, and ethnicity. We propose a cost-effective ancillary study that will leverage existing data from the parent Hispanic Community Health Study/Study of Latinos (HCHS/SOL) while collecting new data on sexual orientation, gender identity, stigma, discrimination, stress, coping, social support, and CVD risk. The proposed study is timely because HCHS/SOL participants will be scheduled for their third in-person visits (V3) starting in November 2019. In this study, we will (1) examine the influence of sexual orientation and gender identity on CVD risk among all HCHS/SOL participants at V3 (~9300); (2) model pathways from sexual orientation and gender identity to CVD risk through stigma, discrimination, and stress among a 1:2 matched sub-cohort of SGM and non-SGM participants at V3 (~1680); and (3) examine the influence of stigma/discrimination on sexual orientation and CVD risk relationships among sub-cohort participants at V3. Data analysis will follow a conceptual model derived from the LGBT Minority Stress Model in which excess stigma discrimination against SGM leads to minority stress that increases CVD risk. In this model, coping and social support serve as resilience factors that mitigate the impact of minority stress on CVD risk. Cross-sectional and longitudinal regression models as well as structural equation models will be used to test these relationships. Understanding the influence of stigma-induced stress on CVD risk among Hispanic/Latino SGM has significant implications for the development of culturally-specific CVD risk reduction strategies. Study findings will be used to build on identified Hispanic/Latino cultural strengths to inform adaptation and testing of family and community acceptance interventions. .

PUBLIC HEALTH RELEVANCE: The cardiovascular health of Hispanic/Latino sexual and gender minorities has been understudied. The goal of this study is to examine relationships between sexual/gender minority stress, coping, social support, and heart disease - a leading cause of morbidity and mortality among Hispanic/Latinos in the U.S. Findings will inform development of culturally-appropriate interventions to address psychosocial factors that may contribute to cardiovascular health disparities by sexual orientation and gender identity.

CRITIQUE 1

Significance: 2
Investigator(s): 1

Innovation: 3
Approach: 2
Environment: 1

Overall Impact: The proposed study is likely to have high impact on public health science. Score-driving strengths include the strong conceptual foundation, rigor of scientific premise, excellent investigative team, and synergy with ongoing parent study, which makes the new knowledge this study would add relatively cost effective. Minor weaknesses include some ambiguity in the precise operationalization of SGM status in analytic models (as opposed to in screening questions) and the fact that the hypothesized associations are consistent with such a large body of literature on stigma, stress, and CVD. On balance, the addition of data on a cohort of people with multiple minority statuses outweighs this weakness on innovation and promises to add valuable new data on a neglected population.

1. Significance:
Strengths
- Good foundation in Minority Stress Model, with complementary support from framework of intersectionality.
- Strong scientific premise for likelihood of increased vulnerability to CVD among Latino sexual and gender minorities (SGM).
- Elevated CVD among SGMs are a disparity in need of study and intervention.

Weaknesses
- Conceptualization of SGM is not particularly nuanced. The category of SGM masks substantial heterogeneity in sexual orientation, identity, and behavior, and one would expect hypothesized associations to vary along these lines.

2. Investigator(s):
Strengths
- Multiple PIs complement one another well.
- Demonstrated record of collaboration and productivity among HCHS/SOL researchers.
- Strong coverage of multiple disciplines required for the study.
- Excellent record of accomplishment in scientific productivity across the team.

Weaknesses
- None noted.

3. Innovation:
Strengths
- Study adds needed data on intersection of multiple minority identities, notably SGM among Latinos.
- Longitudinal designs remain too uncommon among studies of minority stress and health.
- Measures of stigma as separate from stress add valuable new data.

Weaknesses
- As application argues convincingly, there are already strong reasons to expect that SGM is associated with elevated CVD risk among Latinos.

4. Approach:
Strengths
- Ancillary study builds on successful HCHS/SOL, the largest longitudinal cohort study of US Hispanic/Latinos.
- Large parent cohort study (N=9300 expected for V3) should yield adequate sample of Latinos identifying as SGM (~560).
- Addition of CRP to other cardiometabolic risk markers is valuable.
- Strong link between conceptual model and validated measures for key constructs.

- Good attention to power and sample size
- Consideration of scientific rigor, quality control, and potential problems and alternatives.

Weaknesses

- Data cited concerning feasibility of collecting data on sexual and gender minority identity are not tailored to the cohort. A particular problem is that the evidence cited indicates that “most respondents” do not find such questions difficult or sensitive. But “most” is not the point: Do people who hold the minority status find it difficult or sensitive to report? Given the application’s argument about stigma, there needs to be stronger evidence that stigma wouldn’t prevent stigmatized people from disclosing their identities.
- Argument for screening with a question on sexual attraction is sound, but it is unclear precisely how SGM versus non-SGM will be classified in analyses. Do the researchers expect a difference in effect of stigma or discrimination between people who report same-sex attraction versus those who report identity or behavior?

5. Environment:
Strengths

- Strong intellectual environment with support for interdisciplinary exchange and collaboration.
- Ample research infrastructure, including libraries, computing, and collaboration centers.

Weaknesses

- None noted.

Protections for Human Subjects:
Acceptable Risks and/or Adequate Protections

- Adequate protections.

Data and Safety Monitoring Plan (Applicable for Clinical Trials Only):

Inclusion Plans:

- Sex/Gender: Distribution justified scientifically
- Race/Ethnicity: Distribution justified scientifically
- For NIH-Defined Phase III trials, Plans for valid design and analysis: Not applicable
- Inclusion/Exclusion Based on Age: Distribution justified scientifically
- Youngest cohort members now 26 years old.

Vertebrate Animals:
Not Applicable (No Vertebrate Animals)

Biohazards:
Acceptable

- Specific biohazards are not addressed, but the team has experience collecting and protecting biomarker data.

Authentication of Key Biological and/or Chemical Resources:
Not Applicable (No Relevant Resources)

Budget and Period of Support:
Recommend as Requested

CRITIQUE 2

Significance: 2
Investigator(s): 1
Innovation: 1
Approach: 2

Environment: 1

Overall Impact: The proposed study intends to develop an ancillary project to the existing SOL study to examine relationships between CVD risk factor and sexual orientation, gender identify, stigma, discrimination, stress, coping and social support. Cross-sectional and longitudinal analyses will be used to tests the proposed relationships. Key strengths of the application include; strong scientific premise and clear public health significance to conduct this ancillary study. Excellent team of co-PIs and co-Is with the appropriate methodological and conceptual expertise to conduct the proposed study. Innovative population, strong theoretical model and a rigorous study design with validated measures and clear analytical plan for each of the study hypothesis. No major weaknesses were identified in this proposal. Overall, this is a significant, innovative and rigorous study that intends to address a critical gap in understanding the health of the SGM Latino population in the U.S. This study can make a significant contributions to the fields of SGM health disparities research and Latinx health research.

1. Significance:
Strengths
- This study is significant since cardiovascular health of SGM Latinos has been understudied and this is the fastest growing ethnic group within the SGM population in the U.S.
- A strong scientific premise and theoretical framework supports the proposed study.

Weaknesses
- None noted

2. Investigator(s):
Strengths
- The study Co-PIs bring complementary expertise in SMG health disparities, Latino CVD health, survey methodologies and leading and designing rigorous epidemiological studies. They both have the necessary expertise and experiences to conduct the proposed study.
- A strong team of co-investigators supports the proposed study. This team has a history of previous collaborations and are well connected with the SOL study.
- This is the right team for this innovative study.

Weaknesses
- None noted

3. Innovation:
Strengths
- The examination of the intersection of sexual orientation, gender identity, Latinx ethnicity on CVD risk in a state-of-the-art on-going cohort epidemiological study is novel. Results from this study can make substantial contributions to this important literature.
- The focus on SGM Latinx population is novel given the scarcity of research in this growing and diverse population.
- The proposed longitudinal analyses are also novel.

Weaknesses
- None noted.

4. Approach:
Strengths
- Clear study aims and hypotheses inform the proposed study.
- The proposed study leverages the infrastructure and rigorousness of the existing and ongoing SOL study.
- Measuring stigma and discrimination with well validated measures separately is a key strength of the approach.
- The study builds upon previous analyses using the SOL data to examine the relationships between many of the proposed concepts which inform the proposed analyses.

- A strong theoretical framework supports the proposed analyses.
- Rigorous study procedures to recruit and collect data on the SGM subsample are being used.
- Rigorous and well-validated measures using objective indicators of CVD risk, MetS and CMR will be used in the proposed study.
- Clear analytical plans are presented for each study hypotheses, including primary, secondary and exploratory analyses.

Weaknesses

- If sample size permits, exploratory analysis examining differences between Latinx subgroups (e.g., country of birth, etc) could be considered for each of the study aims.

5. Environment:
Strengths

- Excellent institutional support and research infrastructure supports the proposed study.

Weaknesses

- None noted.

Study Timeline:
Strengths

- Not applicable

Weaknesses

- None noted.

Protections for Human Subjects:
Acceptable Risks and/or Adequate Protections

- Adequate protection

Data and Safety Monitoring Plan (Applicable for Clinical Trials Only):
Not Applicable (No Clinical Trials)

Inclusion Plans:

- Sex/Gender: Distribution justified scientifically
- Race/Ethnicity: Distribution justified scientifically
- For NIH-Defined Phase III trials, Plans for valid design and analysis: Not applicable
- Inclusion/Exclusion Based on Age: Distribution justified scientifically
- All distributions are scientifically justified.

Vertebrate Animals:
Not Applicable (No Vertebrate Animals)

Biohazards:
Not Applicable (No Biohazards)

Resource Sharing Plans:
Acceptable

Authentication of Key Biological and/or Chemical Resources:
Acceptable

Budget and Period of Support:
Recommend as Requested

CRITIQUE 3

Significance: 2
Investigator(s): 1
Innovation: 3
Approach: 3
Environment: 2

Overall Impact: This ancillary study adds new dimensions to Latinx health and CVD studies by focusing on the SGM population. This application makes good use of existing data and adds to the existing literature. The analytic plans are strong, there is a strong team in place. While this is significant, there are weaknesses in the approach that are noted. Another concern is about the start time. If wave 3 starts in November, it is unclear if there will be enough time to prepare for recruiting participants. Overall, this remains a strong study.

1. Significance:
Strengths
- This is ancillary study adds new dimensions to Latinx health and CVD studies by looking at the SGM population which is a high risk, rapidly growing, and experience greater health disparities compared to non-Latino Whites.
- This study provides a unique opportunity to leverage a large cohort study

Weaknesses
- None noted

2. Investigator(s):
Strengths
- This is an MPI team with complementary expertise in SGM health and in Latino health
- Collectively, the team has done extensive research exploring social and structural drivers of health disparities in sexual and gender minorities
- This team has evidence of prior collaborations on relevant publications and previous grants

Weaknesses
- None noted

3. Innovation:
Strengths
- Conducting an ancillary study that adds SOGI questions to a prospective cohort Latinos contributes to the literature
- The study can assess data over 12 years and explore temporal trends

Weaknesses
- There is strong evidence to suggest that SGM will have higher CVD risk, experience greater stigma therefore, the innovative aspect is slightly diminished because of this fact.

4. Approach:
Strengths
- Presents a cost effective opportunity to leverage existing data
- Applies minority stress and intersectionality as frameworks
- Assesses protective factors that may play a role in mitigating stigma
- They will use same data collection as the parent study
- The inclusion of measures on connectedness, affirmation, and belonging will add to understanding of pathways by which stigma leads to increased CVD risk

Weaknesses:
- These are not major weaknesses and they can be clarified
- It is not clear how “any history of same sexual attraction” will be analyzed or will this be included in the same category as SGM

- Do individuals who report “any history of same sexual attraction” experience the same risks and stigma and do they identify themselves as SGM
- Another concern is that visit 3 starts in Nov 2019 which is a short time frame
- A more detailed discussion on how inflammatory markers (HsCRP) are affected would strengthen the background and lend greater support for the proposed analyses

5. Environment:
Strengths
- Strong environment that is built on strong research foundation

Weaknesses
- None noted

Protections for Human Subjects:
Data and Safety Monitoring Plan (Applicable for Clinical Trials Only):
Acceptable

Inclusion Plans:
- Sex/Gender: Distribution justified scientifically
- Race/Ethnicity: Distribution justified scientifically
- For NIH-Defined Phase III trials, Plans for valid design and analysis:
- Inclusion/Exclusion Based on Age: Distribution justified scientifically

Vertebrate Animals:
Not Applicable (No Vertebrate Animals)

Biohazards:
Not Applicable (No Biohazards)

Authentication of Key Biological and/or Chemical Resources:
Not Applicable (No Relevant Resources)

Budget and Period of Support:
Recommend as Requested

THE FOLLOWING SECTIONS WERE PREPARED BY THE SCIENTIFIC REVIEW OFFICER TO SUMMARIZE THE OUTCOME OF DISCUSSIONS OF THE REVIEW COMMITTEE, OR REVIEWERS' WRITTEN CRITIQUES, ON THE FOLLOWING ISSUES:

PROTECTION OF HUMAN SUBJECTS: ACCEPTABLE

INCLUSION OF WOMEN PLAN: ACCEPTABLE

INCLUSION OF MINORITIES PLAN: ACCEPTABLE

INCLUSION ACROSS THE LIFESPAN PLAN: ACCEPTABLE

COMMITTEE BUDGET RECOMMENDATIONS: The budget was recommended as requested.

Footnotes for 1 R01 HL149778-01; PI Name: POTEAT, TONIA C.

NIH has modified its policy regarding the receipt of resubmissions (amended applications). See Guide Notice NOT-OD-14-074 at http://grants.nih.gov/grants/guide/notice-files/NOT-OD-14-074.html. The impact/priority score is calculated after discussion of an application by averaging the overall scores (1-9) given by all voting reviewers on the committee and multiplying by 10. The criterion scores are submitted prior to the meeting by the individual reviewers assigned to an application, and are not discussed specifically at the review meeting or calculated into the overall impact score. Some applications also receive a percentile ranking. For details on the review process, see http://grants.nih.gov/grants/peer_review_process.htm#scoring.

MEETING ROSTER

Health Disparities and Equity Promotion Study Section
Healthcare Delivery and Methodologies Integrated Review Group
CENTER FOR SCIENTIFIC REVIEW
HDEP
06/20/2019 - 06/21/2019

Notice of NIH Policy to All Applicants: Meeting rosters are provided for information purposes only. Applicant investigators and institutional officials must not communicate directly with study section members about an application before or after the review. Failure to observe this policy will create a serious breach of integrity in the peer review process, and may lead to actions outlined in NOT-OD-14-073 at https://grants.nih.gov/grants/guide/notice-files/NOT-OD-14-073.html and NOT-OD-15-106 at https://grants.nih.gov/grants/guide/notice-files/NOT-OD-15-106.html, including removal of the application from immediate review.

CHAIRPERSON(S)

BOUTIN-FOSTER, CARLA, MD
ASSOCIATE DEAN
OFFICE OF DIVERSITY EDUCATION AND RESEARCH
SUNY DOWNSTATE MEDICAL CENTER
BROOKLYN, NY 11203

MEMBERS

ALLEN, HEIDI LYNN, PHD
ASSOCIATE PROFESSOR
SCHOOL OF SOCIAL WORK
COLUMBIA UNIVERSITY
NEW YORK, NY 10027

ARRIOLA, KIMBERLY, PHD
PROFESSOR
DEPARTMENT OF BEHAVIORAL SCIENCES AND
HEALTH EDUCATION
ROLLINS SCHOOL OF PUBLIC HEALTH OF
EMORY UNIVERSITY
ATLANTA, GA 30322

CABASSA, LEOPOLDO J, PHD
ASSOCIATE PROFESSOR
BROWN SCHOOL OF SOCIAL WORK
WASHINGTON UNIVERSITY IN ST. LOUIS
ST. LOUIS, MO 63130

CUNNINGHAM, PHILLIPPE BELTON, PHD *
PROFESSOR
DEPARTMENT OF PSYCHIATRY AND BEHAVIORAL SCIENCE
CENTER FOR GLOBAL AND COMMUNITY HEALTH
FAMILY SERVICES RESEARCH CENTER
MEDICAL UNIVERSITY OF SOUTH CAROLINA
MEDICAL UNIVERSITY OF SOUTH CAROLINA
CHARLESTON, SC 29401

DESAI, JAY R, PHD
RESEARCH INVESTIGATOR
HEALTH PARTNERS INSTITUTE
BLOOMINGTON, MN 55425

GARCIA, VICTOR Q, PHD
PROFESSOR AND DIRECTOR
MID-ATLANTIC RESEARCH AND
TRAINING INSTITUTE FOR COMMUNITY
AND BEHAVIORAL HEALTH
INDIANA UNIVERSITY OF PENNSYLVANIA
INDIANA, PA 15705

GIURGESCU, CARMEN, PHD *
ASSOCIATE PROFESSOR
COLLEGE OF NURSING
THE OHIO STATE UNIVERSITY
COLUMBUS, OH 43210

GRAVLEE, CLARENCE C, PHD
ASSOCIATE PROFESSOR
DEPARTMENT OF ANTHROPOLOGY
UNIVERSITY OF FLORIDA
GAINESVILLE, FL 32611

JAHNKE, SARA ANNE, PHD *
PRINCIPAL INVESTIGATOR
NATIONAL DEVELOPMENT AND
RESEARCH INSTITUTES
LEAWOOD, KS 66224

JONES, WARREN ALLEN, MD *
DISTINGUISHED PROFESSOR OF HEALTH POLICY
DEPARTMENT OF MINORITY MEN'S HEALTH INITIATIVE
HAMPTON UNIVERSITY
HAMPTON, VA 23668

KARNIK, NIRANJAN, MD, PHD
VICE CHAIR PSYCHIATRY
DEPARTMENT OF PSYCHIATRY
RUSH UNIVERSITY MEDICAL CENTER
CHICAGO, IL 60612

KO, LINDA K, PHD *
ASSOCIATE PROFESSOR
FRED HUTCHINSON CANCER RESEARCH CENTER
UNIVERSITY OF WASHINGTON
SEATTLE, WA 98195

MEYER, ILAN H, PHD *
WILLIAMS DISTINGUISHED SENIOR SCHOLAR FOR PUBLIC POLICY
THE WILLIAMS INSTITUTE
SCHOOL OF LAW
UNIVERSITY OF CALIFORNIA, LOS ANGELES
LOS ANGELES, CA 90095

NATTINGER, ANN B MPH, MD *
SENIOR ASSOCIATE DEAN FOR RESEARCH
PROFESSOR OF MEDICINE
MEDICINE AND HEALTH SERVICES RESEARCH
MEDICAL COLLEGE OF WISCONSIN
MILWAUKEE, WI 53226

POLIVKA, BARBARA J, PHD, BSN *
ASSOCIATE DEAN OF RESEARCH AND PROFESSOR
SCHOOL OF NURSING
UNIVERSITY OF KANSAS MEDICAL CENTER
KANSAS CITY, KS 66160

QUINN, GWENDOLYN P, PHD
ENDOWED PROFESSOR
DEPARTMENT OF OBSTETRICS AND GYNECOLOGY
DEPARTMENT OF POPULATION HEALTH
SCHOOL OF MEDICINE
NEW YORK UNIVERSITY
NEW YORK, NY 10016

ROSENZWEIG, MARGARET Q, PHD
PROFESSOR
DEPARTMENT OF ACUTE/TERTIARY CARE
SCHOOL OF NURSING
UNIVERSITY OF PITTSBURGH
PITTSBURGH, PA 15261

ROSS, LEVI A, PHD *
ASSOCIATE PROFESSOR
DEPARTMENT OF HEALTH SCIENCE
COLLEGE OF HUMAN ENVIRONMENTAL SCIENCES
THE UNIVERSITY OF ALABAMA
TUSCALOOSA, AL 35487

SALES, ANNE EVELYN, PHD *
PROFESSOR
DEPARTMENT OF LEARNING HEALTH SCIENCES
SCHOOL OF MEDICINE
UNIVERSITY OF MICHIGAN
ANN ARBOR, MI 48109

SHALOWITZ, MADELEINE ULLMAN, MD
DIRECTOR OF BIOMEDICAL RESEARCH INFORMATICS
ASSOCIATE PROFESSOR
DEPARTMENT OF PEDIATRICS
UNIVERSITY OF CHICAGO
EVANSTON, IL 60201

SHUMWAY, MARTHA, PHD
ASSOCIATE PROFESSOR
DEPARTMENT OF PSYCHIATRY
SCHOOL OF MEDICINE
UNIVERSITY OF CALIFORNIA SAN FRANCISCO
SAN FRANCISCO, CA 94143

SINGH, PRAMIL NAND, DRPH *
DIRECTOR AND ASSOCIATE PROFESSOR
CENTER FOR HEALTH RESEARCH
LOMA LINDA UNIVERSITY
LOMA LINDA, CA 92350

SOULAKOVA, JULIA, PHD *
ASSOCIATE PROFESSOR
BURNETT SCHOOL OF BIOMEDICAL SCIENCES
UNIVERSITY OF CENTRAL FLORIDA
ORLANDO, FL 32827

TANJASIRI, SORA P, DRPH, MPH
PROFESSOR
DEPARTMENT OF EPIDEMOLOGY
UNIVERSITY OF CALIFORNIA, IRVINE
IRVINE, CA 92697

VERNEY, STEVEN P, PHD *
ASSOCIATE PROFESSOR
DEPARTMENT OF PSYCHOLOGY
ALBUQUERQUE, NM 87131

VIDRINE, JENNIFER IRVIN, PHD
PROFESSOR
OKLAHOMA TOBACCO RESEARCH CENTER
STEPHENSON CANCER CENTER
DEPARTMENT OF FAMILY AND PREVENTIVE MEDICINE
UNIVERSITY OF OKLAHOMA HEALTH SCIENCES CENTER
OKLAHOMA CITY, OK 73104

WENDEL, MONICA L, DRPH *
ASSOCIATE DEAN FOR PUBLIC HEALTH PRACTICE
ASSOCIATE PROFESSOR, HEALTH PROMOTION & BEH. SCI.
DIRECTOR, COMMONWEALTH INSTITUTE OF KENTUCKY
SCHOOL OF PUBLIC HEALTH AND INFORMATION SCIENCES
UNIVERSITY OF LOUISVILLE
LOUISVILLE, KY 40202

MAIL REVIEWER(S)

WU, BEI, PHD
DEAN'S CHAIR PROFESSOR
RORY MEYERS COLLEGE OF NURSING
NEW YORK UNIVERSITY
NEW YORK , NY 10010

SCIENTIFIC REVIEW OFFICER

BELLINGER, JESSICA, PHD
SCIENTIFIC REVIEW ADMINISTRATOR
CENTER FOR SCIENTIFIC OF REVIEW
NATIONAL INSTITUTES OF HEALTH
BETHESDA, MD 20892

EXTRAMURAL SUPPORT ASSISTANT

NJOKU, PHILIP C
EXTRAMURAL SUPPORT ASSISTANT
DIVISION OF AIDS, BEHAVORIAL, POPULATION SCIENCES
HEALTHCARE DELIVERY AND METHODOLOGIES (HDM)
NATIONAL INSTITUTES OF HEALTH
BETHESDA, MD 20892

* Temporary Member. For grant applications, temporary members may participate in the entire meeting or may review only selected applications as needed.

Consultants are required to absent themselves from the room during the review of any application if their presence would constitute or appear to constitute a conflict of interest.
